# Drug storage, polypharmacy and frailty syndrome in older people: an observational study

**DOI:** 10.11604/pamj.2023.45.29.32636

**Published:** 2023-05-11

**Authors:** Maturin Tabué-Téguo, Roxane Villeneuve, Jeannie Helene-Pelage, Matteo Cesari, Jordane Chovino, Axelle Boire, Moustapha Dramé, Nadine Simo-Tabué, Denis Boucaud-Maitre

**Affiliations:** 1 Centre Hospitalo-Universitaire de Guadeloupe, Pointe-à-Pitre, Guadeloupe, France,; 2 Laboratoire de Mathématiques-Informatique et Applications (LAMIA), Université des Antilles, Pointe-à-Pitre, Guadeloupe, France,; 3 Institut National de la Santé et de la Recherche Médicale (*INSERM U*1219), Université de Bordeaux, Bordeaux, France,; 4 Département de Médecine Générale, Université des Antilles, Pointe-à-Pitre, Guadeloupe, France,; 5 Geriatric Unit, Istituto di Ricovero e Cura a Carattere Scientifico di natura pubblica (IRCCS) Ca´ Granda Ospedale Maggiore Policlinico, Milano, Italy,; 6 Centre Hospitalo-Universitaire de Martinique, Université des Antilles, Fort-de-France, Martinique, France

**Keywords:** Frailty, polypharmacy, drug storage, elderly

## Abstract

**Introduction:**

the increasing prevalence of polypharmacy in the older population could lead to inappropriate storage of medicines at home. Since polypharmacy is associated with frailty, the main objective of the Karukera Study of Aging - Drug Storage (KASADS) study was to investigate the association between drug storage and frailty. If such an association exists, drug storage could be a simple tool for the identification of medication vulnerability by non-medical staff in the elderly.

**Methods:**

observational, cross-sectional study in community-dwelling older adults (>65 years old). Drug storage was defined as any drug in excess compared to a medical prescription, any unused and/or expired drug, or any drug without a medical prescription. Frailty was measured with the Study of Osteoporotic Fractures (SOF) scale, and polypharmacy was defined as a prescription of at least 5 drugs. Bivariate and multivariate analyses were performed to study the associations between drugs storage, frailty, and polypharmacy.

**Results:**

during the study period (01/10/2019 to 15/03/2020), 115 elderly people were interviewed in their own homes. The average age was 76.0 ± 7.8 years old. Seventy-two percent of the participants met the criteria for polypharmacy and 30.4% were prefrail/frail. They stored an average of 14.7 ± 18.2 boxes. Drug storage was associated with polypharmacy (17.5 boxes versus 10.0; p=0.031) but not with frailty (15.6 versus 14.3; p=0.724). In multivariate analysis, drug storage was associated with not having a school degree (OR: 1.78; 95%CI: 1.13-2.79), suffering from dyslipidemia (OR: 2.00; 95% CI: 1.28-3.17) and suffering from cognitive disorders evaluated by the Mini Mental State Examination (MMSE) score (OR: 1.10; 95%CI: 1.02-1.17).

**Conclusion:**

drug storage was not significantly associated with frailty. Nevertheless, it was associated with polypharmacy and other medical outcomes, and could therefore represent a new area for research in geriatrics and pharmacy.

## Introduction

For several years now, in France, public authorities have been launching mass media campaigns encouraging users to return unused medicines to pharmacies. These messages were aimed at tackling self-medication, and especially iatrogenesis and its deleterious effects among the older population [1], partly stemming from inappropriate storage of medicines at home. However, drug storage has received little attention in the scientific community and has no consensual definition. In an Australian study [2], conducted in 2018 in 166 households, the authors found 1429 “unused” drugs. Storage was defined as “any medicine not used daily” (unused medicines and those with an expiry date that had passed). In another study in Pakistan, assessing the causes of drug storage and the different uses of the stored medicines [3], drug storage was not clearly defined. However, according to the authors, 100% of households “stocked” drugs: current treatment represented 20.4% of the stored medicine; 70.9% were drugs kept for future use, and 8.7% were leftover drugs from previous treatments. Over the last decades, the number of drugs per prescription has increased [4], and this could be one of the main reasons for the inappropriate storage of medicines at home. However, the link between polypharmacy and drug storage has seldom been studied.

Polypharmacy is defined by the World Health Organization (WHO) as “the administration of many drugs at the same time or the administration of an excessive number of drugs” [5-7]. As is the case for a majority of geriatric syndromes, there is as yet no consensus on its definition. A threshold of 5 or more drugs is commonly reported [8,9]. The prevalence of polypharmacy increases with age, from 55.9% in people in their forties to 88.9% in octogenarians. It then decreases in centenarians (26.3%). It is therefore a public health issue for older people. Moreover, the relationship between polypharmacy and the risk of negative health events in older people has been clearly demonstrated, such as falls, dependency, nursing home admission, death, etc., and particularly frailty [10].

**Figure 1:**
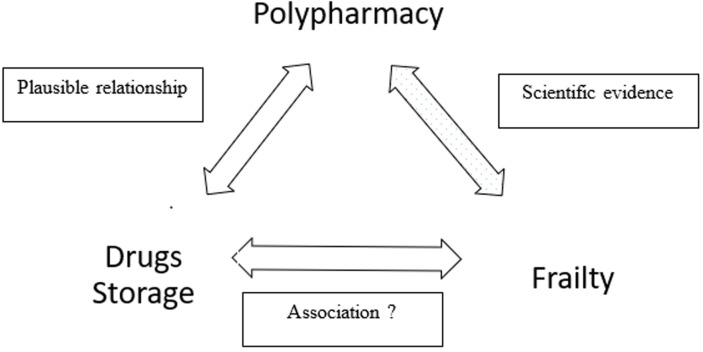
main hypothesis of the study

Drug storage, polypharmacy, and frailty are all parameters associated with aging and could be interrelated. The association between frailty and drug storage needs to be explored, as drug storage could serve as a simple tool (requiring no medical expertise) for the identification of frailty by non-medical staff in the older population (Figure 1). However, to our knowledge, no study has assessed the relationship between frailty and drug storage, and there are relatively few reports on the link between polypharmacy and drug storage. The main objective of the KASADS study was therefore to investigate an association between drug storage and frailty syndrome in the elderly (Figure 1).

## Methods

**Study design:** this prospective, observational, cross-sectional study was conducted in Guadeloupe (French West Indies) between October 1, 2019, and March 15, 2020. The study was approved by the Ethics Committee of the University Hospital of Guadeloupe (Ref: A6_19_10_01_KASADS).

**Setting and participants:** the participants of the study were recruited from the patient base of 15 volunteer GPs in the territory. Within the same week, each physician had to systematically recommend the study to older people consulting at their medical practice, until the quota of 10 older people per physician was reached. The inclusion criteria were as follows: man or woman aged at least 65 years old, affiliated to a social security plan, with a medical prescription at the end of the visit. The criteria for non-inclusion were refusal to participate in the study, severe or unstable general pathology, being under guardianship, or living in an institution.

The selected patients were contacted by phone by two medical school students, and after obtaining a verbal agreement, the same two medical school students went to their homes to collect data, using a standard case report form.

**Variables:** the following variables were collected: age, gender, medical history, level of education, current and previous prescriptions, frailty, undernutrition, cognitive status, and dependence assessment scales. All the drugs that were stored, in the bathroom or in another room of the house, were recorded.

Drug storage was defined as: any drug in excess of the medical prescription prescribed by the various doctors, including those used on a daily basis; not used, and/or expired; as well as any non-prescription drug. Storage was analyzed according to the total number of boxes (main analysis), the total number of boxes under the list, and the total number of boxes over the counter. When the storage concerned the same drug, all the boxes were counted. Frailty was assessed using the SOF scale [11,12] based on the following 3 questions: (1) unintentional weight loss in the last year of more than 5 kilos; (2) ability to get up from a chair 5 times without using the arms; (3) lack of energy in the last week. Each item was scored as “1” or “0”, a score of 0 corresponding to a robust older person, a score of 1 or more was considered frail.

Polypharmacy was defined as a prescription for at least 5 medications [8]. Participants were asked whether they were diagnosed with hypertension, dyslipidemia, and diabetes. Disability for the basic activities of daily living [13] (ADL) and instrumental (IADL) was assessed based on the Katz index and Lawton and Brody scale [14] respectively. The Katz index is designed to assess the subject's capacity to perform ADL such as taking a bath, getting dressed, and using the toilet among others. On the other hand, the Lawton and Brody scale evaluates the subject's ability to perform IADLs such as using the phone, managing money, using transportation, and shopping, among others. Other scales were used to assess nutritional status Mini Nutritional Assessment Short-Form (MNA-SF) [15], and cognitive status (MMSE) [16].

**Study size:** no sample size was calculated, due to the lack of literature on drug storage. Nevertheless, the intended sample size was 150 patients in this exploratory study.

**Statistical methods:** quantitative variables were expressed as mean ± standard deviation, categorical variables as percentages. Bivariable analyses were conducted to compare 1. The clinical characteristics and the amount of medicine packages in storage between patients with frailty or no frailty syndrome 2. The association between the amount of medicine packages in storage and polypharmacy. For the multivariable analysis, a quasi-Poisson model was used to study the relationship between the amount of medicine packages in storage and the variables of interest (age, sex, education, hypertension, diabetes, dyslipidemia, frailty, MMSE, and IADL scores). P-values, a threshold of 0.05, and 95% confidence intervals were reported. All analyses were performed using R v.3.0.2.

## Results

**Participants:** a total of 115 older people were included out of the 150 originally planned. The inclusion of patients was stopped on March 15, 2020 due to the COVID-19 crisis and the ensuing lockdown in France [17]. These participants were 76.0 ± 7.8 years old on average, were predominantly female (67.8%), and had a BMI of 26.8 ± 5.3 kg/m^2^. In our sample, 43.5% suffered from diabetes, 87.0% from hypertension, 58.3% from obesity, and 52% from dyslipidemia. Polypharmacy was observed in 63% of the participants (Table 1). The prevalence of drug storage was 82.6% (95 out of 115 people were storing drugs). A feature of prefrailty or frailty was observed in 35 (30.4%) older people, and these frail people were older than the robust (79.5 years versus 74.5, p=0.001) and more often on polypharmacy (77.1% versus 56.6%) (Table 1).

**Table 1:** characteristics of participants by frailty profile

Characteristics	Total sample	Frailty syndrome at baseline		
	N=115 (±SD or %)	Yes (n=35)	No (n=80)	P-value
Age (years)	76.0 ± 7.8	79.5 ± 8.7	74.5 ± 7.0	0.001
Gender (men)	37 (32.2%)	13 (37.1%)	24 (30.0%)	0.451
BMI (kg/m^2^)	26.8 ± 5.3	27.2 ± 6.9	26.6 ± 4.6	0.673
Living alone (n and % yes)	67 (58.3%)	22 (62.9%)	45 (56.2%)	0.508
Education (without diploma)	39 (33.9%)	18 (51.4%)	21 (26.2%)	0.009
Diabetes	50 (43.5%)	15 (42.9%)	35 (43.7%)	0.929
Hypertension	100 (87.0%)	32 (91.4%)	68 (85.0%)	0.346
Dyslipidémia	52 (45.2%)	18 (51.4%)	34 (42.5%)	0.376
Disability >1IADL	3.4 ± 1.0	2.6 ± 1.4	3.7 ± 0.6	<0.001
Disability >1ADL	5.1 ± 1.0	4.8 ± 1.2	5.3 ± 1.8	0.58
MMSE score	23.7 ± 3.6	21.9 ± 3.8	24.5 ± 3.1	<0.001
Polymedication	72 (62.6%)	27 (77.1%)	45 (56.2%)	0.033

IADL: instrumental activity of daily living; ADL: activity of daily living; BMI: body mass index; MMSE: mini mental state examination

### Main results

**Figure 2:**
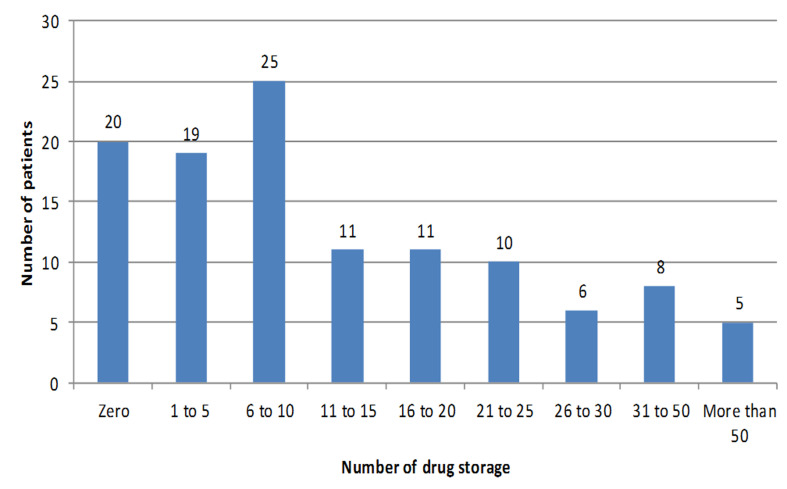
number of drug storage

**Associations between drug storage, frailty and polypharmacy:** a total of 1,696 boxes of medicine were registered, with an average of 14.7 ± 18.2 boxes per older person. Only 17.4% of older people did not stock any medication. The distribution for storage is shown in Figure 2. No difference was observed between frail and robust older people on the total number of packages (15.6 versus 14.3; p=0.724), the total number of packages under prescription (8.3 versus 7; p=0.566), or the total number of packages over the counter (7.3 versus 7.4; p=0.945) (Table 2).

**Table 2:** relationship between drug storage, frailty and polypharmacy

		Frailty syndrome			Polypharmacy		
Medicine packages in storage	Total sample	Yes (n=35)	No (n=80)	p	Yes (n=72)	No (n=43)	p
Total number of packages	14.7 ± 18.2	15.6 ± 16.8	14.3 ± 18.9	0.724	17.5 ± 19.6	10.0 ± 14.6	0.031
Number of packages - prescription drugs	7.4 ± 11.1	8.3 ± 11.7	7.0 ± 11.9	0.566	9.2 ± 11.6	4.3 ± 9.2	0.021
Number of packages - over-the-counter drugs	7.4 ± 9.1	7.3 ± 7.3	7.4 ± 9.8	0.945	8.3 ± 9.8	5.9 ± 7.5	0.168

On the other hand, polypharmacy was associated with storage with respect to the total number of boxes (17.5 versus 10.0; p=0.031) and the number of prescription boxes (9.2 versus 4.3; p=0.021) (Table 2).

### Other analyses

**Determinants of drug storage:** in a multivariate analysis (Table 3), overall drug storage was associated with not having a degree (OR: 1.78; 95%CI: 1.13-2.79), being dyslipidemic (OR: 2.00; 95% CI: 1.28-3.17) and MMSE score (OR: 1.10; 95%CI: 1.02-1.17).

**Table 3:** determinants of drug storage

	Total number of packages		Number of packages - prescription drugs		Number of packages - over-the-counter drugs	
Variables	OR [CI95%]	p	OR [CI95%]	p	OR [CI95%]	p
Age	0.98 [0.95-1.01]	0.067	0.96 [0.93-1.00]	0.067		0.753
Gender (men)	1.50 [0.93-2.38]	0.010	2.02 [1.18-3.39]	0.010		0.561
Living alone	1.48 [0.95-2.33]	0.087	1.50 [0.92-2.54]	0.109	1.50 [0.91-2.45]	0.109
Level of education (no degree)	1.78 [1.13-2.79]	0.019	1.52 [0.92-2.34]	0.012	1.43 [0.89-2.32]	0.150
Diabetes	0.89 [0.59-1.37]	0.628	0.74 [0.45-1.21]	0.242		0.631
Hypertension	0.81 [0.43-1.64]	0.370	0.68 [0.31-1.68]	0.370		0.533
Dyslipidemia	2.00 [1.28-3.17]	0.003	2.67 [1.57-4.78]	<0.001	1.63 [1.02-2.64]	0.046
Frailty (robust)	1.01 [0.60-1.59]	0.963	1.06 [0.61-1.91]	0.838		0.940
Polypharmacy	1.61 [0.98-2.71]	0.068	1.98 [1.10-3.75]	0.031		0.360
MMSE	1.10 [1.02-1.17]	0.009	1.10 [1.01-1.19]	0.026	1.11 [1.03-1.19]	0.009
IADL	0.93 [0.70-1.24]	0.620	0.86 [0.63-1.20]	0.371		0.935

IADL: instrumental activity of daily living; MMSE: mini-mental state examination

Prescription drug storage was associated with male sex (OR: 2.02; 95%CI: 1.18-3.39), being dyslipidemic (OR: 2.67; 95%CI: 1.57-4.78), being on polypharmacy (1.98; 95%CI: 1.10-3.75) and MMSE score (OR: 1.10; 95%CI: 1.01-1.19). Finally, storage of over-the-counter drugs was only associated with the MMSE score (OR: 1.11; 95%CI: 1.03-1.19) and with being dyslipidemic (OR: 1.63; 95%CI: 1.02-2.64) (Table 3).

## Discussion

In this study, we did not find any significant association between drug storage and frailty in older people, for both prescription and over-the-counter drugs. Storage is therefore not a potential tool for detecting frailty, unlike, for example, reduced walking speed, which seems to be a reliable tool for identifying the risk of dependence, institutionalization, falls or mortality [18,19]. Nevertheless, this tool is not necessarily applied in routine care, and remains poorly accessible to untrained people.

From a clinical point of view, this lack of association between drug storage and frailty remains intriguing. The expected results were indeed in favor of a strong association, in relation to multiple prescriptions, polypharmacy, or non-systematic taking of treatments, particularly in people with low MMSE scores. However, this is not the result that appears in this study.

Nevertheless, our study suggests that drug storage may be associated with other factors. As expected, drug storage was associated with polypharmacy, both for the total number of drugs and the sub-sample of prescription drugs. This large amount of medicines in storage, and the high proportion of people stockpiling drugs, is worthy of further investigation. Beyond the issue of non-prescription drugs, whose storage is facilitated by their accessibility, other hypotheses can be put forward, such as the irregular taking of drugs but their regular renewals or changes in treatment - following hospitalization, changes in health status, discontinuation of a given drug (for example, prescribed by both the general practitioner and a specialist).

One hypothesis that should be explored is the specificity of the drug delivery system in France. Indeed, while in some countries the exact dose of capsules or tablets is delivered to the patient, in France, medicines are only available in packages with a predefined number of tablets [20]. Although the number of tablets is generally calculated to correspond to a weekly or monthly standard treatment plan, it is not uncommon for the number of tablets needed for treatment to be too large. Leftover treatment may be stored in the medicine cabinet, “just in case” instead of being taken back to the pharmacy. This accumulation of unused medicines also has a significant cost for social security, as the quantities charged are higher than the quantities prescribed. For example, Megerlin [21,22], and Lhoste [23,24] estimated the cost of medicines prescribed, but not used, in nursing homes at €60,409,310.40 per year (for 574,670 residents). Moreover, the French healthcare system facilitates access to medicines (free of charge for the patient in a majority of cases). This situation favors stockpiling for both frail and non-frail people. This may explain the lack of significant difference.

Since drug storage and polypharmacy are associated, further studies would be needed to determine whether drug storage is associated with other health events related to polypharmacy, including non-adherence to treatment, iatrogenesis falls [25], hospitalization, and mortality [26,27].

**Limitations:** the positive association between drug storage and MMSE score may seem counter-intuitive at first glance. However, the lower the MMSE score, the more likely it is that older people are no longer responsible for refilling prescriptions at the pharmacy. In people with cognitive impairment, if medication management is overseen by an informal caregiver or healthcare professional, risks of buying unnecessary non-prescription drugs are lessened, and proper renewal and intake of medication altogether are more likely. Nevertheless, no information is available in our database regarding family and social support, such as the presence of a nurse or a relative to help with medication management. In addition, we cannot rule out that for people with cognitive impairment who do not live alone, some non-prescription medications could be intended for others in the same household. We also found an association between drug storage and dyslipidemia. This result, quite surprising, could be explained in part by the impact of cardiovascular risk factors (diabetes, hypertension, obesity…) in polypharmacy in older persons and those the due to the association with other chronic diseases and/or complications.

## Conclusion

Drug storage is an understudied topic, even though it could provide a simple tool for detecting negative health events, in order to implement preventive action. In our study, drug storage was not associated with frailty, but larger studies would be necessary to confirm or infirm our results. Drug storage was nevertheless associated with polypharmacy, which is itself associated with negative health events (iatrogenicity, hospitalization, etc.) that could be worth exploring in the context of future research.

### What is known about this topic

- Polypharmacy in the older population could lead to inappropriate storage of medicines at home;
- Since polypharmacy is associated with frailty, the main objective of this study was to investigate the association between drug storage and frailty.

### What this study adds

- Drug storage was not associated with frailty but associated with other clinical outcomes;
- Drug storage is an understudied topic, even though it could provide a simple tool for detecting negative health events, in order to implement preventive actions;
- Drug storage could therefore represent a new area for research in geriatrics and pharmacy.
